# miR‐375‐3p/STX6 Exacerbates Atherosclerosis by Promoting Endothelial Cell Senescence via Activation of TGF‐Beta Signals

**DOI:** 10.1111/acel.70326

**Published:** 2025-12-10

**Authors:** Ying Zhu, Zhirui Liu, Yiqi Wan, Shuangjin Ding, Jiankun Liu, Andong Wu, Ximo Dai, Jin Zhou, Xueer Li, Xueting Gong, Man Liu, Xiao‐Li Tian

**Affiliations:** ^1^ Human Aging Research Institute (HARI) and School of Life Science Nanchang University, and Jiangxi Province Key Laboratory of Aging and Disease Nanchang Jiangxi China; ^2^ Department of Blood Transfusion First Affiliated Hospital of Gannan Medical University Ganzhou Jiangxi China; ^3^ Key Laboratory of Prevention and Treatment of Cardiovascular and Cerebrovascular Diseases, Ministry of Education Gannan Medical University Ganzhou Jiangxi China; ^4^ State Key Laboratory of Experimental Hematology, National Clinical Research Center for Blood Diseases, Institute of Hematology & Blood Diseases Hospital Chinese Academy of Medical Sciences & Peking Union Medical College Tianjin China; ^5^ Haihe Laboratory of Cell Ecosystem Tianjin China; ^6^ Tianjin Institutes of Health Science Tianjin China; ^7^ Institute of Molecular Medicine, College of Future Technology Peking University Beijing China

**Keywords:** atherosclerosis, endothelial, miR‐375‐3p, senescence, STX6

## Abstract

Atherosclerosis, a key pathological basis of cardio‐cerebrovascular diseases, is closely associated with aging and endothelial cell senescence. The role of microRNAs (miRNAs) in regulating endothelial cell senescence and atherosclerosis remains incompletely understood. In this study, we discovered that miR‐375‐3p expression was significantly elevated in the serum of both aged and atherosclerotic mice. Overexpression of miR‐375‐3p induced endothelial cell senescence, evidenced by increased senescence‐associated β‐galactosidase (SA‐β‐gal) staining, upregulation of p15, IL6, and IL8, and inhibited cell colony formation. In vivo inhibition of miR‐375‐3p in ApoE^−/−^ mice markedly reduced atherosclerotic plaque formation. We further identified STX6 as a direct target of miR‐375‐3p, and its overexpression rescued the senescence‐related phenotypes induced by miR‐375‐3p. Mechanistically, the miR‐375‐3p/STX6 signaling axis promoted endothelial cell senescence via the SMAD2/p15 pathway in a SMAD2‐dependent manner, and overexpression of STX6 attenuated atherosclerosis progression in mice. Together, our findings highlight the miR‐375‐3p/STX6 axis as a critical regulator of endothelial cell senescence and a potential translational use in the prevention of atherosclerosis and related diseases.

## Graphical abstract

miR‐375‐3p promotes endothelial senescence by targeting STX6 and activating the SMAD2/p15 pathway (penal A). Inhibition of miR‐375‐3p or restoration of STX6 alleviates endothelial senescence and reduces atherosclerotic plaque formation in ApoE^−/−^ mice (penal B).

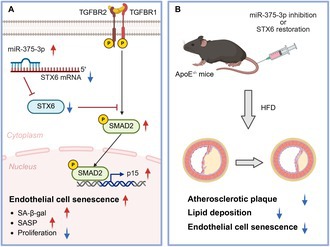

## 1 Introduction

Atherosclerosis is a complex, multifactorial inflammatory process. It is marked by the formation of atherosclerotic plaques on arterial walls, with aging being a major risk factor (Libby et al. 2019). Epidemiological studies have shown that the prevalence of atherosclerosis rises steeply with age, rising from 4.5% in individuals aged 20s to 51.2% in those over 60s (Sturlaugsdottir et al. 2016). Vascular aging is a key determinant in the development of atherosclerosis (Li et al. 2023; Tian and Li 2014), and endothelial cell senescence, along with endothelial dysfunction, is recognized as an initial step in its pathogenesis (Ramírez et al. 2022; Xu et al. 2021). Therefore, elucidating the molecular mechanisms underlying endothelial cell senescence is of significant importance for the development of novel and effective therapies for atherosclerosis.

MicroRNAs (miRNAs) are small non‐coding RNAs that inhibit gene expression by mainly binding to complementary sequences of 3′UTR in their target mRNAs (Mohr and Mott 2015; Saliminejad et al. 2019). Existing studies suggest that miRNAs are involved in a variety of biological processes such as cell development, differentiation, autophagy, and apoptosis (Sanuki and Yamamura 2021; Sun et al. 2020; Wu et al. 2018). Recent studies of several miRNAs, such as miR‐21, miR‐217 (Mensà et al. 2020), miR‐34a (Guo et al. 2017), and miR‐146a (Xiao et al. 2021), have identified them as modulators of the senescent process of endothelial cells, suggesting the importance of epigenetic regulation in endothelial cell senescence.

miR‐375‐3p is known as a tumor suppressor miRNA that inhibits the proliferation of cancer cells (Li et al. 2021; Xu et al. 2020; Zhang et al. 2018; Zou et al. 2017). In addition to its role in cancer, other investigations have reported that miR‐375‐3p serves as a biomarker for diabetes and plays essential roles in maintaining the normal function of pancreatic β‐cells (Latreille et al. 2015; Poy et al. 2009). Notably, miR‐375‐3p level is increased with age; for example, in the serum of healthy Chinese individuals (Zhang et al. 2015) and in DOX‐induced senescent K562 cells (Yang et al. 2012), and importantly, miR‐375‐3p is involved in the impairment of vascular functions (Mao et al. 2021; Shi et al. 2021; Zhang et al. 2021). It has been demonstrated that the silencing of miR‐375‐3p modulates macrophage polarization, attenuates foam cell formation, and inhibits the progression of atherosclerosis by targeting Krüppel‐like factor 4 (KLF4) (Chen, Li, et al. 2019; Qiu et al. 2021). Nevertheless, whether miR‐375‐3p regulates endothelial cell senescence and, in turn, contributes to the development of atherosclerosis remains largely unexplored.

In this study, we identified miR‐375‐3p as a novel regulator of endothelial cell senescence in atherosclerosis. We demonstrated that miR‐375‐3p is significantly upregulated in the serum of aged and atherosclerotic mice, and its in vivo inhibition alleviated atherosclerotic lesion formation, revealing that miR‐375‐3p promotes endothelial cell senescence by targeting STX6 and activating the SMAD2/p15 signaling pathway. Our findings elucidate a previously unrecognized role of miR‐375‐3p in endothelial cell senescence and suggest its potential as a translational target for atherosclerosis.

## 2 Results

### 2.1 Age‐Related miR‐375‐3p Promotes Endothelial Cell Senescence

To identify those miRNAs that are differentially expressed in endothelial senescence, firstly, we used our senescent miRNA database to screen out 210 candidate miRNAs conserved in rat, mice, and human that may be associated with senescence (Figure S1, Table S1). Next, the mimics of these 210 candidate miRNAs were synthesized and transfected into HUVECs. The SA‐β‐gal staining assay was performed to identify the 26 miRNAs that could significantly induce endothelial cell senescence (Table S2). Among these, miR‐375‐3p emerged as a strong candidate, as suggested by its consistent association with SA‐β‐gal positive endothelial cells.

**FIGURE 1:**
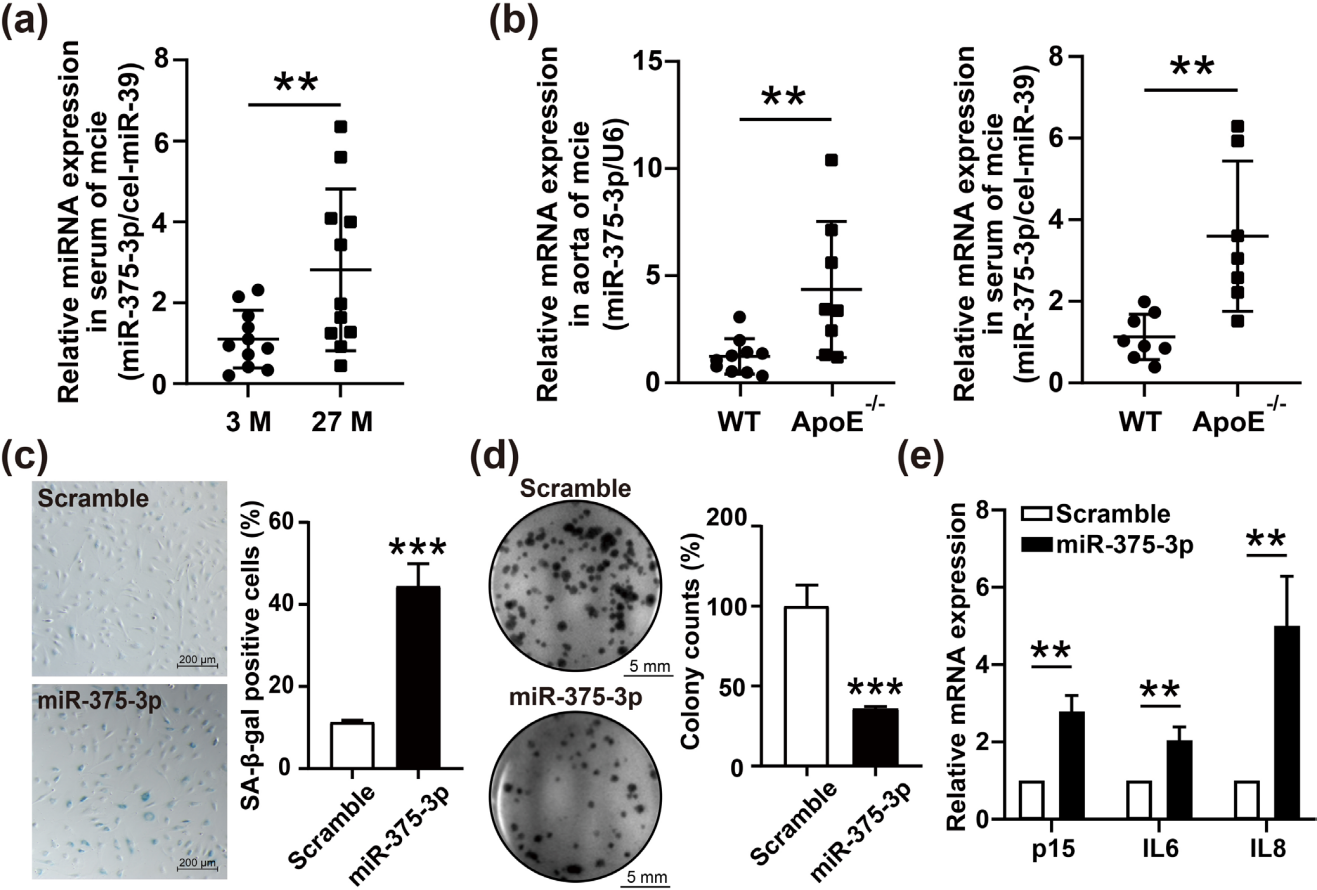
Age‐increased miR‐375‐3p promotes endothelial cell senescence. (a) qRT‐PCR analysis of miR‐375‐3p expression in serum from young (3 months old, *n* = 11) and aged (27 months old, *n* = 11) mice, normalized to cel‐miR‐39. M, months. (b) miR‐375‐3p expression in aortas (WT, *n* = 10; ApoE^−^/^−^, *n* = 8) and serum (WT, *n* = 8; ApoE^−^/^−^, *n* = 7) of mice fed a HFD for 8 weeks, normalized to cel‐miR‐39 or U6. (c) Representative images and quantification of SA‐β‐gal staining in HUVECs transfected with miR‐375‐3p mimic or scramble. Scale bar = 200 μm. (d) Representative images and quantification of colony formation assay showing cell proliferation in HUVECs transfected with miR‐375‐3p mimic or scramble. Scale bars = 5 mm. (e) qRT‐PCR analysis of p15, IL6, and IL8 mRNA expression in HUVECs transfected with miR‐375‐3p mimic or scramble, normalized to GAPDH. Data are presented as mean ± SD; statistical significance was determined by unpaired two‐tailed Student's *t*‐test. *p < 0.05, **p < 0.01, ***p < 0.001.

We examined the expression of miR‐375‐3p in the serum of young (3 months old) and aged (27 months old) mice. Our results showed that miR‐375‐3p was significantly upregulated in the serum of aged mice compared to young mice (Figure 1a). To further investigate the association between miR‐375‐3p and atherosclerosis, we evaluated its expression in the aorta and serum of wild‐type (WT) and ApoE‐deficient (ApoE^−/−^) mice fed a high‐fat diet (HFD) for 8 weeks. We found that miR‐375‐3p was significantly increased in both the aorta and serum of ApoE^−/−^ mice compared to WT mice (Figure 1b).

To investigate whether miR‐375‐3p contributes to endothelial cell senescence, we performed a SA‐β‐gal staining assay. The results showed that the percentage of SA‐β‐gal positive cells was significantly higher in HUVECs with overexpression of miR‐375‐3p compared to the scramble control group (Figure 1c). Additionally, in the colony formation assay, HUVECs overexpressing miR‐375‐3p exhibited significantly fewer colonies, indicating a marked reduction in proliferative capacity (Figure 1d). Furthermore, p15, a key cell cycle inhibitor associated with senescence, and IL6 and IL8, major components of the senescence‐associated secretory phenotype (SASP), were all upregulated at the mRNA level by miR‐375‐3p (Figure 1e). These findings suggest that age‐related upregulation of miR‐375‐3p promotes endothelial cell senescence.

### 2.2 In Vivo Inhibition of miR‐375‐3p Alleviates Endothelial Senescence and Atherosclerosis

Given that miR‐375‐3p expression is markedly elevated with age and promotes endothelial senescence in vitro, we next sought to determine whether miR‐375‐3p functionally contributes to vascular aging and atherosclerosis development in vivo. To this end, we performed in vivo inhibition experiments using a miR‐375‐3p antagomir in ApoE^−/−^ mice fed a high‐fat diet. Mice received weekly tail vein injections of the miR‐375‐3p antagomir (50 nmol/20 g body weight) for 6 weeks (Figure S2a). Consistent with our in vitro findings, suppression of miR‐375‐3p markedly attenuated endothelial senescence, as indicated by decreased SA‐β‐gal positive staining in the aortic root (Figure S2d). Moreover, Oil Red O staining revealed a significant reduction in atherosclerotic plaque burden in the entire aortic tree (Figure S2b). In the aortic root, both lesion area and lipid‐positive staining showed a downward trend following miR‐375‐3p inhibition, although the latter did not reach statistical significance (*p* = 0.1857; Figure S2c). Collectively, these data demonstrate that miR‐375‐3p promotes endothelial senescence and accelerates atherosclerosis development in vivo, while its inhibition mitigates vascular aging and lesion formation.

### 2.3 STX6 Is a Target of miR‐375‐3p in Endothelial Cell Senescence

12 potential downstream target genes of miR‐375‐3p were screened (Figure S3a) by comprehensive analysis of 149 reported predicted potential downstream target genes (Cao et al. 2013) and our previous data (RNA sequencing data of DOX/MMC induced and replicative senescence in HUVECs). After dual luciferase reporter assay, 4 genes (YAP1, STX6, RIDA, PAFAH1B1) were selected (Figure S3b,c). Except for YAP1 (has been reported), RIDA (cannot rescue the cellular senescence which is induced by miR‐375‐3p, Figure S3d,f,h) and PAFAH1B1 (the knockdown in HUVECs with no induction of cellular senescence, Figure S3e,g), we focused on STX6.

**FIGURE 2:**
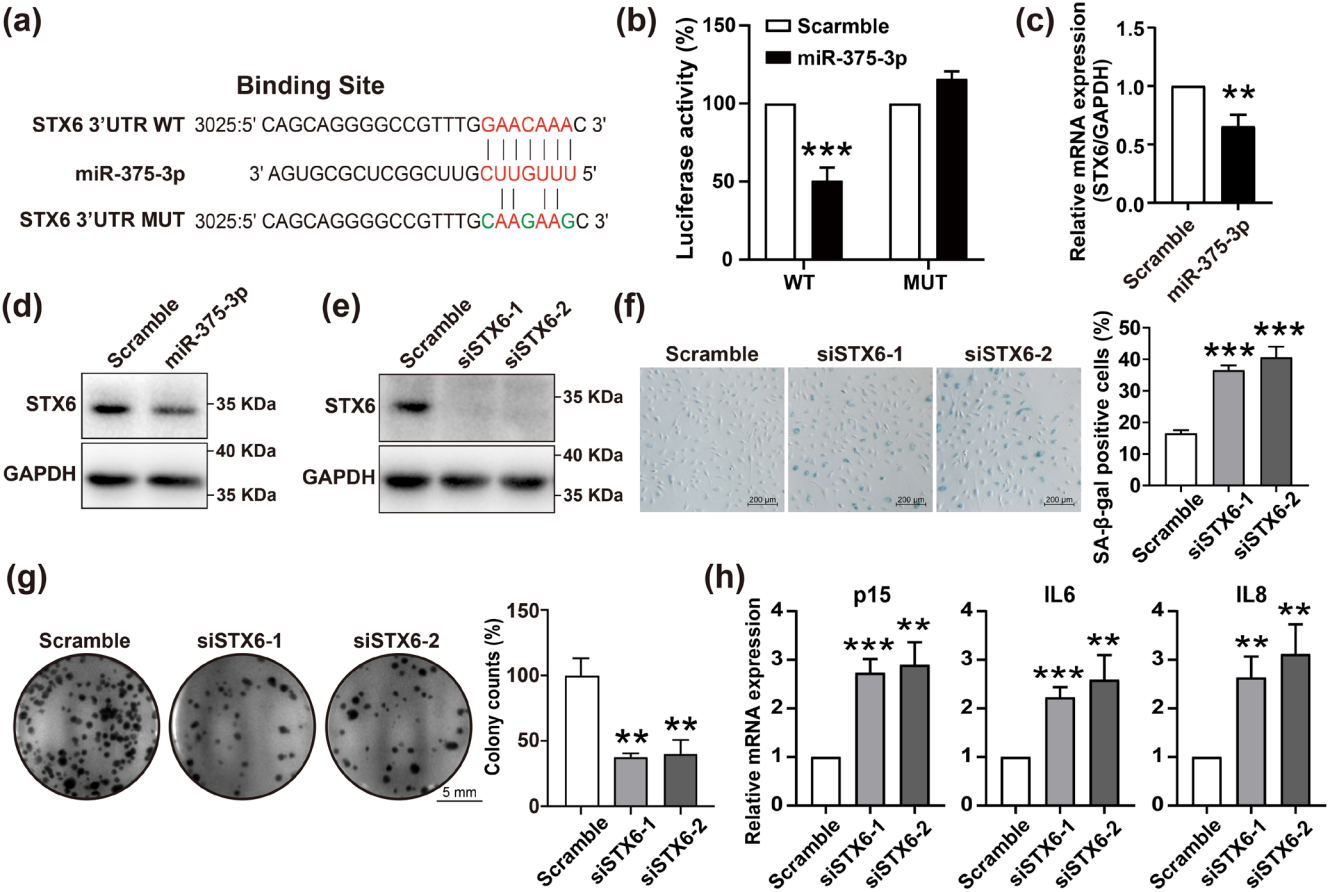
STX6 is a target of miR‐375‐3p in endothelial cell senescence. (a) Predicted and mutated miR‐375‐3p binding sites within the 3′UTR of STX6. (b) Luciferase assay of WT and mutant STX6 3′UTR reporters in HEK293A cells transfected with miR‐375‐3p mimic or scramble. (c) qRT‐PCR analysis of STX6 mRNA in HUVECs transfected with miR‐375‐3p mimic or scramble, normalized to GAPDH. (d, e) Western blot analysis of STX6 protein expression in HUVECs transfected with miR‐375‐3p mimic (d) or STX6 siRNAs (e), normalized to GAPDH. (f) Representative SA‐β‐gal staining and quantification in HUVECs transfected with STX6 siRNAs or scramble. Scale bar = 200 μm. (g) Representative colony formation images showing cell proliferation in HUVECs transfected with STX6 siRNAs or scramble. Scale bar = 5 mm. (h) qRT‐PCR analysis of p15, IL6, and IL8 mRNA in HUVECs transfected with STX6 siRNAs or scramble, normalized to GAPDH. Data are presented as mean ± SD; statistical significance determined by unpaired two‐tailed Student's *t*‐test. **p < 0.01, ***p < 0.001.

STX6, a membrane trafficking regulator, was found to have a binding site for miR‐375‐3p in its 3′UTR (Figure 2a), and was verified to be the direct target of miR‐375‐3p by dual luciferase assay. The fragments of STX6 3′UTR containing the predicted (wild‐type, WT) or mutant (MUT) miR‐375‐3p binding site were cloned into the pMIR‐REPORT luciferase vector; miR‐375‐3p exerted a 50% inhibitory effect on the luciferase activity in the construct that contains the WT sequence of the miR‐375‐3p binding site, but showed no inhibitory effect on the mutated miR‐375‐3p binding site (Figure 2b). Additionally, qRT‐PCR and western blot showed that the expression of STX6 was down‐regulated in HUVECs after miR‐375‐3p overexpression (Figure 2c,d). These results revealed that miR‐375‐3p specifically and directly suppressed STX6 expression by binding with the STX6 3′UTR.

Colony formation count and SA‐β‐gal staining rate were compared between STX6 siRNAs‐treated and scramble group to understand whether STX6 plays a regulatory role in endothelial cell senescence. Figure 2e showed the knockdown efficiency of STX6 siRNAs. Knockdown of STX6 significantly increased the positive ratio of SA‐β‐gal staining rate (Figure 2f) and inhibited HUVEC proliferation (Figure 2g). Also, the mRNA expression of p15, IL6, and IL8 was up‐regulated upon STX6 knockdown (Figure 2h), which is consistent with the result from miR‐375‐3p overexpression. Our results demonstrate that STX6 is a direct target of miR‐375‐3p in promoting endothelial cell senescence.

### 2.4 STX6 Overexpression Rescues the Pro‐Senescent Effect of miR‐375‐3p

**FIGURE 3:**
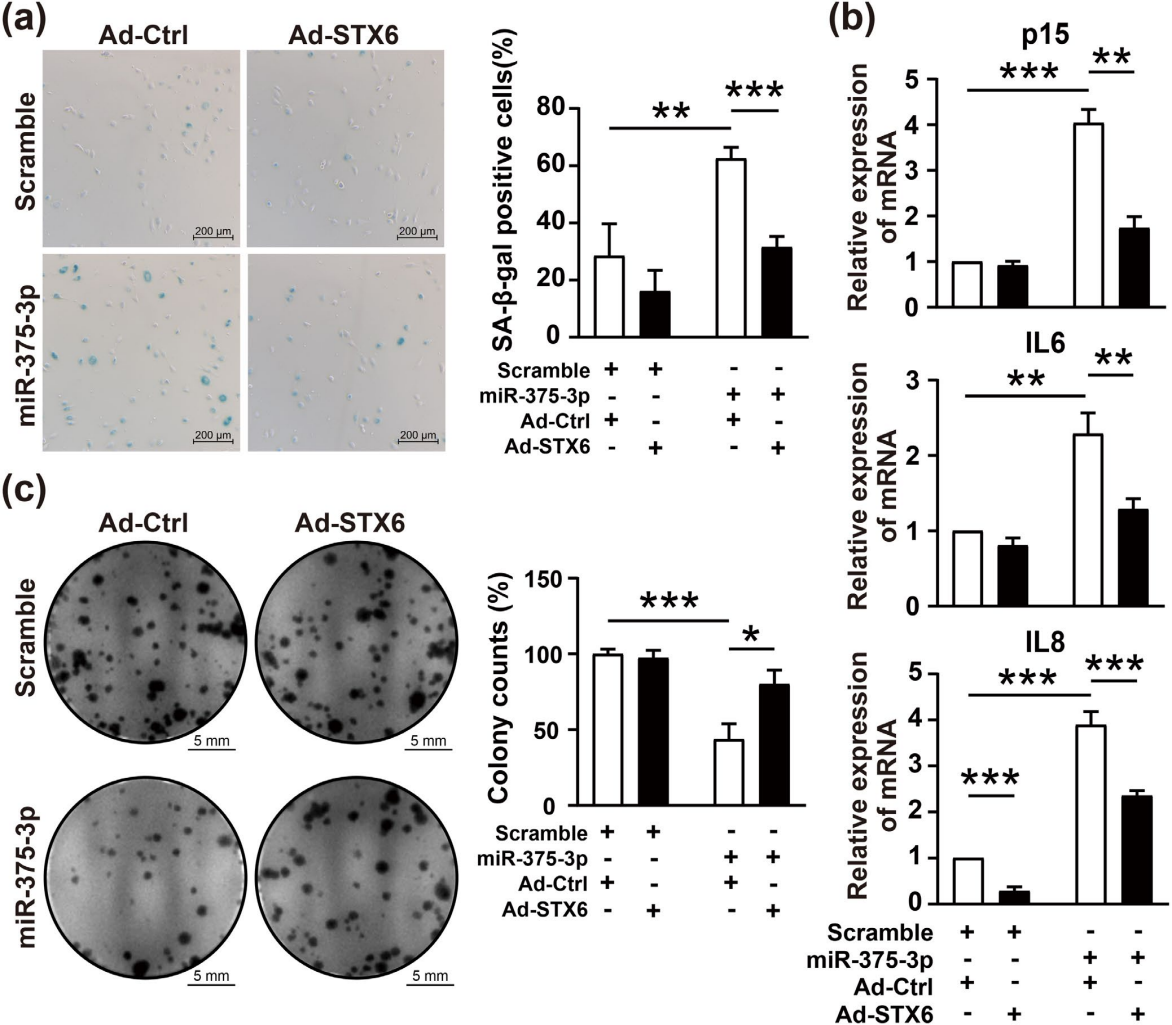
STX6 overexpression rescues the pro‐senescent effect of miR‐375‐3p. (a) Representative SA‐β‐gal staining and quantification in HUVECs infected with Ad‐STX6 and transfected with miR‐375‐3p mimic. Scale bar = 200 μm. (b) qRT‐PCR analysis of p15, IL6, and IL8 mRNA expression in HUVECs infected with Ad‐STX6 and transfected with miR‐375‐3p mimic, normalized to GAPDH. (c) Colony formation assay showing cell proliferation in HUVECs infected with Ad‐STX6 and transfected with miR‐375‐3p mimic. Scale bar = 5 mm. Data are presented as mean ± SD; statistical significance determined by two‐way ANOVA. *p < 0.05, **p < 0.01, ***p < 0.001.

As STX6 has been confirmed to be the direct target of miR‐375‐3p, further investigation was performed to study whether overexpression of STX6 could rescue the senescence‐related phenotypes induced by miR‐375‐3p. STX6 was successfully overexpressed by recombinant adenovirus vector (Figure S4). The pro‐senescent effect of miR‐375‐3p was partially abrogated by STX6 overexpression (Figure 3a). Meanwhile, STX6 overexpression abrogated the increased mRNA expressions of p15, IL6, and IL8 induced by miR‐375‐3p (Figure 3b). The overexpression of STX6 rescued the decreased proliferation induced by miR‐375‐3p overexpression (Figure 3c). These validated that miR‐375‐3p/STX6, as a signaling cascade, contributed to endothelial cell senescence.

### 2.5 miR‐375‐3p/STX6 Signaling Promotes Endothelial Cell Senescence Through the Increased Internalization of TGFBR1

RAB5A mediates the internalization of TGFBR1 stimulated by TGF‐β to EEA‐positive endosomes (He et al. 2015). Previous studies suggested that STX6 was competitively binding to EEA1 and caused the dissociation between EEA1 and RAB5A (Simonsen et al. 1999). Therefore, STX6 may be involved in the suppressive regulation of TGFBR1 internalization and SMAD2 phosphorylation.

**FIGURE 4:**
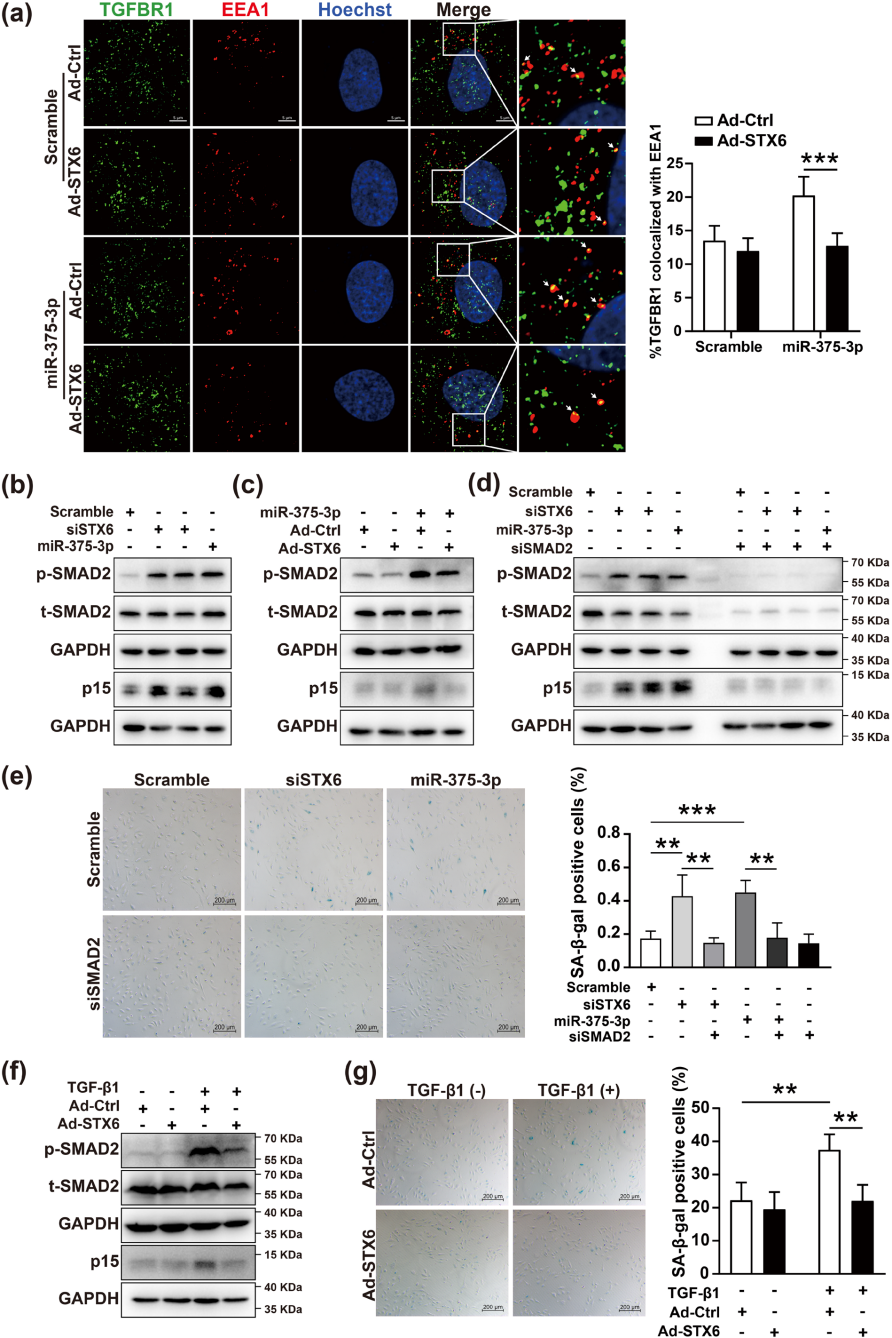
miR‐375‐3p/STX6 signaling promotes endothelial cell senescence through the increased internalization of TGFBR1. (a) Immunofluorescence images showing colocalization (yellow) of TGFBR1 (green) and EEA1 (red) in HUVECs infected with Ad‐STX6 and transfected with miR‐375‐3p. Nuclei stained with Hoechst (blue). Scale bar = 5 μm. (b–d) Western blot analysis of SMAD2 phosphorylation and p15 expression in HUVECs transfected with miR‐375‐3p mimic or STX6 siRNAs (b), infected with Ad‐STX6 and transfected with miR‐375‐3p mimic (c), or co‐transfected with SMAD2 siRNA and miR‐375‐3p mimic or STX6 siRNAs (d). (e) Representative SA‐β‐gal staining and quantification in HUVECs co‐transfected with SMAD2 siRNA and miR‐375‐3p mimic or STX6 siRNAs. Scale bar = 200 μm. (f, g) Western blot (f) and SA‐β‐gal (g) staining in HUVECs infected with Ad‐STX6 and stimulated with TGF‐β1. Scale bar = 200 μm. Data are presented as mean ± SD; statistical significance determined by unpaired two‐tailed Student's *t*‐test or two‐way ANOVA. **p < 0.01, ***p < 0.001.

Immunofluorescence showed that the increased internalization of TGFBR1 was induced upon miR‐375‐3p overexpression and overexpression of STX6 restrained this process (Figure 4a), which demonstrated that miR‐375‐3p/STX6 signaling promotes endothelial cell senescence through regulating the internalization of TGFBR1. Previous research showed that the SMAD2 phosphorylation was dependent on TGF‐β receptor internalization (Penheiter et al. 2002). Therefore, we selected the phosphorylation level of SMAD2 to characterize TGFBR1 internalization. As shown in Figure 4b, miR‐375‐3p overexpression or STX6 knockdown significantly increased the phosphorylation level of SMAD2 and the protein expression of p15. Moreover, miR‐375‐3p induced activation of SMAD2/p15 signaling was able to be restricted by STX6 overexpression (Figure 4c).

To confirm whether miR‐375‐3p/STX6 signaling plays its role in a SMAD2‐dependent manner, miR‐375‐3p mimic or STX6 siRNAs were co‐transfected with SMAD2 siRNA in HUVEC, respectively. The results showed that SMAD2 knockdown significantly delayed HUVEC senescence and inhibited the increase of SMAD2 phosphorylation and p15 expression induced by miR‐375‐3p overexpression or STX6 knockdown (Figure 4d,e).

SMAD2/p15 signaling pathway is one of the most important pathways induced by TGF‐β1 (Hannon and Beach 1994; Lyu et al. 2018). To elucidate the role of STX6 in TGF‐β1‐induced endothelial cell senescence, we first determined the optimal dose of TGF‐β1 for inducing senescence in HUVECs. Recombinant human TGF‐β1 was added to the culture medium of HUVECs at various concentrations. Consistent with previous studies, we found that higher concentrations of TGF‐β1 (100–200 ng/mL) significantly increased the positive ratio of SA‐β‐gal staining and the protein expression of p15 (Figure S5). Next, to investigate the involvement of STX6 in the TGF‐β1‐mediated activation of the SMAD2/p15 signaling pathway, we overexpressed STX6 in HUVECs. We observed that the STX6 overexpression significantly attenuated the increased positive ratios in SA‐β‐gal staining, SMAD2 phosphorylation, and p15 protein expression which were induced by TGF‐β1 (Figure 4f,g).

Together, these results demonstrated that miR‐375‐3p/STX6 induces endothelial cell senescence via activation of the SMAD2/p15 signaling pathway by the internalization of TGFBR1.

### 2.6 Overexpression of STX6 Reduce Atherosclerotic Plaque in Mice

**FIGURE 5:**
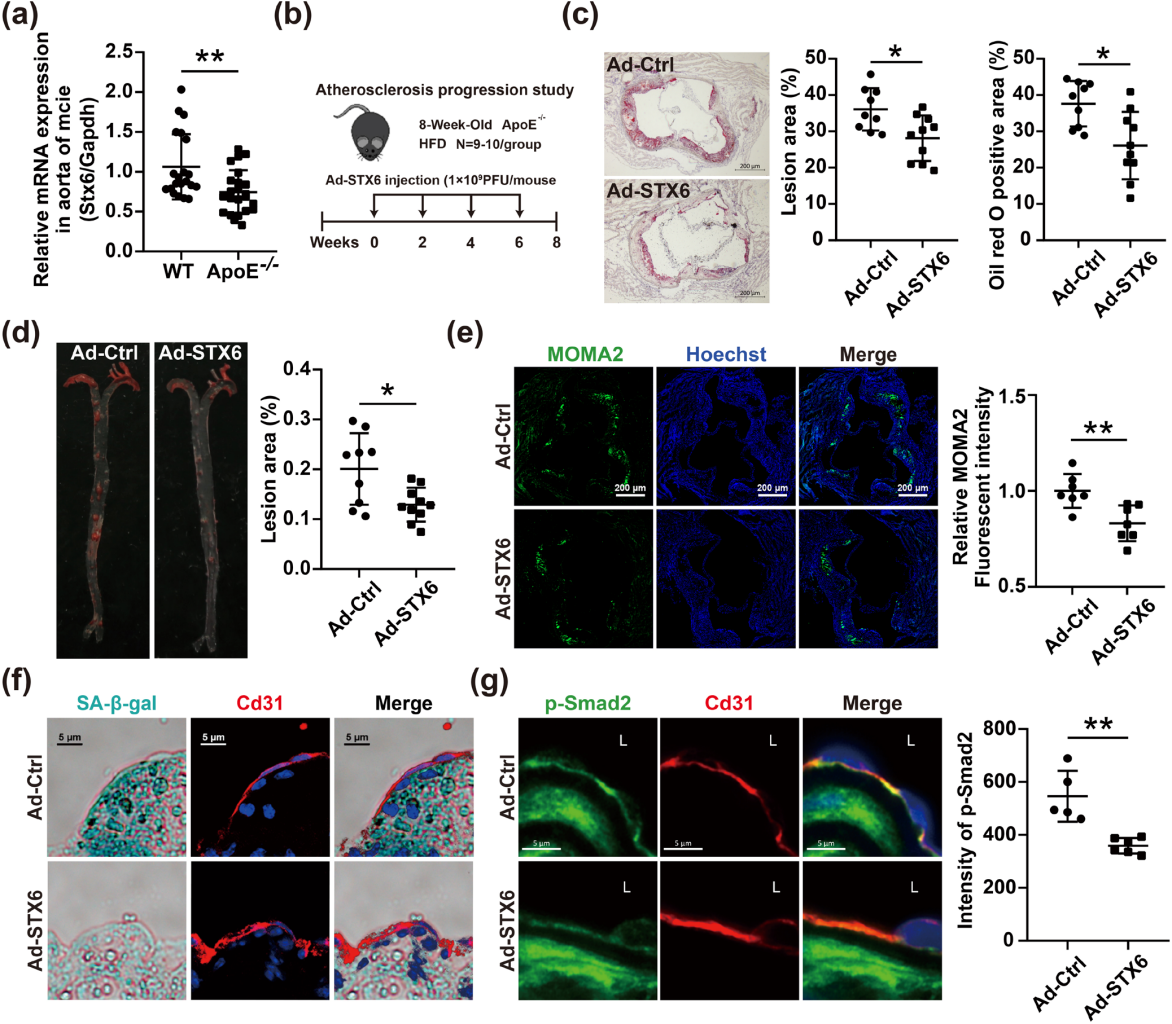
Overexpression of STX6 reduce atherosclerotic plaque in mice. (a) qRT‐PCR analysis of Stx6 expression in aortas of WT (*n* = 21) and ApoE^−/−^ (*n* = 23) mice fed HFD for 8 weeks, normalized to Gapdh. (b) Schematic of experimental design showing Ad‐Ctrl or Ad‐STX6 injection (1 × 10^9^ PFU per mouse, twice weekly for 8 weeks) in ApoE^−/−^ mice on HFD. (c) Representative Oil Red O staining images and quantification of lesion size and positive area in the aortic sinus. Scale bar = 200 μm. (d) Representative Oil Red O staining images and quantification of plaque area in the entire aortic tree. (e) Immunofluorescence staining of macrophage marker MOMA2 (green) in aortic sinus. Scale bar = 200 μm. (f) Representative SA‐β‐gal and Cd31 co‐staining in aortic sinus lesions. Scale bar = 5 μm. (g) Immunofluorescence of phosphorylated Smad2 (green) and Cd31 (red) in the aortic intima (L). Nuclei stained with Hoechst (blue). Scale bar = 5 μm. Data are presented as mean ± SD; statistical significance determined by unpaired two‐tailed Student's *t*‐test. *p < 0.05, **p < 0.01.

To explore the role of the miR‐375‐3p/STX6 signaling axis in atherosclerosis, we examined the expression of STX6 in the aortas of WT and ApoE^−/−^ mice fed a HFD for 8 weeks. We found that STX6 expression was significantly reduced in the aortas of ApoE^−/−^ mice compared to WT mice (Figure 5a). Subsequently, 8‐week‐old ApoE^−/−^ mice were intravenously injected with either Ad‐Ctrl or Ad‐STX6 twice weekly for 8 weeks while on the HFD (Figure 5b). After 8 weeks, adenovirus‐mediated overexpression of STX6 significantly increased its expression in the aortic intima (Figure S6a,b). We further assessed general metabolic characteristics, including body weight and serum lipid profiles. The results showed that total cholesterol (TC) and high‐density lipoprotein cholesterol (HDL‐C) levels were significantly decreased in the Ad‐STX6 group compared with the Ad‐Ctrl group, whereas body weight exhibited no significant change (Figure S6c–e). Notably, Oil Red O staining analysis revealed that Ad‐STX6 treatment mice had a significant decrease in lipid deposition in the aortic sinus and atherosclerotic plaques (Figure 5c), along with a significant decrease in atherosclerotic lesion area across the entire aorta tree (Figure 5d) compared to Ad‐Ctrl. In parallel, immunofluorescence analysis of the aortic root demonstrated that macrophage infiltration, indicated by the mean staining intensity of the macrophage marker MOMA2, was markedly reduced in the Ad‐STX6 group compared with controls (Figure 5e). Additionally, STX6 overexpression decreased the positive ratio of SA‐β‐gal staining and reduced the phosphorylation level of Smad2 in the aortic intima of ApoE^−/−^ mice, thereby validating our in vitro findings (Figure 5f,g). Collectively, these results demonstrate that STX6 plays a critical role in mitigating the initiation and progression of atherosclerosis.

## 3 Discussion

Vascular endothelial cell senescence is one of the major risk factors for cardio‐cerebral vascular diseases (Li et al. 2023; Tian and Li 2014). In this study, we demonstrate that aging‐increased miR‐375‐3p directly binds to STX6 mRNA, promoting endothelial cell senescence by an activation of SMAD2/p15 signaling through STX6‐mediated internalization of TGFBR1, while the block of miR‐375‐3p‐initiated signaling pathway prevents endothelial cell senescence and ameliorates atherosclerosis. Thus, the miR‐375‐3p/STX6‐activated SMAD2/p15 signaling axis may be an important target in the prevention of vascular aging and relevant diseases.

miR‐375‐3p is known as a tumor suppressor and decreased in multiple cancers. Accumulating evidence reported that miR‐375‐3p overexpression suppresses proliferation and triggers apoptosis in both glioma and gastrointestinal stromal tumor (GIST) cells (Gyvyte et al. 2020; Zhang et al. 2018). Some studies focused on the relationship between miR‐375‐3p and autophagy (Kumar et al. 2020; Li et al. 2020). For example, a study indicated that miR‐375‐3p inhibited autophagy and aggravated knee osteoarthritis by targeting ATG2B, an autophagy‐related gene (Li et al. 2020). Another study reported that miR‐375‐3p promotes senescence of pancreatic cancer (PC) cells while circHIF‐1α ameliorates senescence and exacerbates growth in PC cells by sponging miR‐375‐3p (Hua et al. 2025), however, this conclusion needs to be tested in primary cells.

HUVECs is a commonly used cell model to investigate the regulation of endothelial functions (Kandhaya‐Pillai et al. 2022), and that is why we used HUVECs to reveal the effect of miR‐375‐3p on cell senescence in this study. Our results demonstrated that miR‐375‐3p expression was increased in the serum of aged mice. Overexpression of miR‐375‐3p induced a premature senescence‐related phenotype and increased inflammatory response. Similar to the results presented here, miR‐375‐3p has been previously shown to have a significantly increased expression in DOX‐induced senescent K562 cells (Yang et al. 2012). These studies suggested that miR‐375‐3p may play the same role as a senescent promoter in various cell types.

Some mechanisms by which miR‐375‐3p regulates gene expression are already known (Liu et al. 2021), but it is unknown which targets mediate cellular senescence. Here, from the reported potential target genes of miR‐375‐3p (Cao et al. 2013), we identified and functionally confirmed that STX6 was a direct target gene of miR‐375‐3p, as indicated by the luciferase assay and western blot analysis. Further functional studies verified that miR‐375‐3p promoted endothelial cell senescence by directly targeting STX6 in HUVECs. Therefore, our present study discovered for the first time that STX6 is a new target of miR‐375‐3p associated with cellular senescence in HUVECs.

In addition to STX6, YAP1 has also been reported as an important downstream effector of miR‐375‐3p in endothelial cells and atherosclerosis‐related processes (Angom et al. 2022; Barettino et al. 2024; Chao et al. 2021). These studies collectively support the regulatory role of miR‐375‐3p in endothelial senescence and vascular dysfunction. Our findings expand the current understanding of miR‐375‐3p signaling and suggest that both the miR‐375‐3p/STX6 and miR‐375‐3p/YAP1 pathways may cooperatively contribute to endothelial dysfunction and atherosclerosis progression. Further studies will be required to elucidate how these two pathways intersect and whether they act synergistically or independently in vascular aging.

STX6 encodes a TGN (trans‐Golgi network)/endosomal SNARE (soluble N‐ethylmaleimide‐sensitive factor‐attachment protein receptor) protein and is involved in the regulation of various membrane trafficking events (West et al. 2021; Xu 2021), which play important roles in the maintenance of angiogenesis (Manickam et al. 2011; Tiwari et al. 2011), tumorigenesis (Kovacheva et al. 2021), and neuro diseases (Sanchez‐Contreras et al. 2018). p53, as a key tumor suppressor, participates in the regulation of the cell cycle and apoptosis (Wang et al. 2015). STX6, regulated by p53 family proteins (p53, p63, p73), is required for cell proliferation and survival of cancer cells (Zhang et al. 2008). But no study yet reported whether STX6 regulates cell senescence. In this study, we demonstrated that miR‐375‐3p promoted endothelial cell senescence by decreasing the translation of STX6.

Endosomal trafficking is involved in the regulation of many important biological processes, such as aging and autophagy (Raudenska et al. 2021; Simoes et al. 2020). RAB5A‐mediated internalization of TGFBR1 to EEA1‐positive early endosomes is important for the activation of SMAD‐dependent TGF‐β signaling (He et al. 2015; Siegert et al. 2018; Wojciech et al. 2018). TGF‐β signaling is a known senescence‐related pathway and is involved in the pathogenesis of age‐related tissue degeneration (Lyu et al. 2018). An earlier report indicated that STX6 interacts directly with the C‐terminal binding site of EEA1, an effector of RAB5A, which overlaps with that of RAB5A and may cause a dissociation between EEA1 and RAB5A (Simonsen et al. 1999). Our results showed that miR‐375‐3p overexpression promoted colocalization of TGFBR1 and EEA1 and activation of the SMAD2/p15 signal, which was abrogated by STX6 overexpression. A recent study showed that the inhibition of TGF‐beta‐Smad2/3 suppresses cellular senescence and pulmonary fibrosis in aged mice (Y. Li et al. 2024). These together emphasize the importance of TGF‐β/SMAD2 in modulation of cell senescence, and we demonstrated that the pro‐senescent effect of the miR‐375‐3p/STX6 signaling axis was exerted by promoting the internalization of TGFBR1 and the activation of the SMAD2/p15 signaling pathway.

Previous investigations reported that miR‐375‐3p in macrophages was important to the initiation and progression of atherosclerosis (Tabas and Bornfeldt 2016). For instance, it has been demonstrated that miR‐375‐3p silencing regulated the polarization of macrophages, attenuated foam cell formation, and the progression of atherosclerosis by targeting KLF4 (Chen, Li, et al. 2019; Qiu et al. 2021). Here, our results indicated that miR‐375‐3p was increased while STX6 was decreased in the aorta of HFD‐ApoE^−/−^ mice. Furthermore, we and others found that serum miR‐375‐3p was increased in humans and murine during aging (Zarecki et al. 2020; Zhang et al. 2015). Consistently, in vivo inhibition of miR‐375‐3p significantly alleviated endothelial senescence and reduced atherosclerotic plaque formation in ApoE^−^/^−^ mice, further supporting its pathogenic role in vascular aging. These findings, together with that miR‐375‐3p induces endothelial cell senescence, indicate that the role of miR‐375‐3p in the atherosclerotic process is likely multilocus or multi‐step. Another study showed that the inhibition of the TGF‐β signaling pathway reduced vascular inflammation and arrested the progression of atherosclerosis in hyperlipidemic mice (Chen, Qin, et al. 2019), supportive of the importance of the miR‐375‐3p/STX6‐activated SMAD2/p15 signaling axis in vascular aging and atherosclerosis.

Our study demonstrated that adenoviral‐mediated STX6 overexpression effectively alleviated endothelial senescence and atherosclerosis in vivo, providing strong short‐term evidence for the therapeutic potential of STX6. Although the adenoviral system used in this study enabled efficient and robust transgene expression, we acknowledge its inherent immunogenicity, which may limit the duration of expression and complicate long‐term safety evaluation. Similar adenoviral systems, such as those employed in inflammation‐related studies (Jiang et al. 2023), have also been reported to trigger immune activation to varying degrees. Nevertheless, adenoviral vectors remain advantageous for short‐term, high‐expression delivery and for accommodating large genetic inserts. In future work, we plan to use delivery platforms with lower immunogenicity, such as adeno‐associated virus (AAV), to achieve more sustained expression and enable long‐term functional and safety assessments.

In the present study, we showed that miR‐375‐3p/STX6‐activated SMAD2/p15 is a novel signaling axis in cellular senescence and vascular diseases; however, the detailed mechanism of how STX6 internalizes TGFBR1 requires more experimental investigations. So far, there are several published literatures reporting the association of circulatory miR‐375‐3p with coronary artery diseases (Carmona‐Maurici et al. 2024; Luo et al. 2024), the association of STX6 with atherosclerosis is still missing thus needs further validation in humans.

In addition, we will further investigate the potential regulatory interplay between miR‐375‐3p and STX6, including whether a negative feedback mechanism exists between them, both in vitro and in vivo, in future studies. Such efforts will help to clarify the upstream and downstream relationships within the miR‐375‐3p/STX6 axis and its broader impact on endothelial homeostasis and atherosclerosis progression.

In conclusion, we provided evidence that the increased miR‐375‐3p promoted endothelial cell senescence by targeting STX6 and leading to SMAD2/p15 signaling activation. Moreover, overexpression of STX6 alleviated the progression of atherosclerosis in ApoE^−/−^ mice. This extends the knowledge of miRNAs' functions in endothelial cell senescence, and provides a potential biomarker and therapeutic target for atherosclerosis.

## 4 Materials and Methods

### 4.1 Cell Culture and Transfection

HUVECs (ScienCell, Carlsbad, CA, USA) between passages 3 and 5 were cultured in Endothelial Cell Medium (ScienCell, Carlsbad, CA, USA) supplemented with 5% FBS, 1% Endothelial Cell Growth Supplement, and 1% penicillin–streptomycin solution at 37°C in a humidified incubator with 5% CO_2_. HEK293A cells were cultured in DMEM (Gibco, Grand Island, NY, USA) containing 10% FBS (Gibco, Grand Island, NY, USA) and 1% Penicillin/Streptomycin (Gibco, Grand Island, NY, USA).

### 4.2 Animals, Atherosclerosis Mice Model and Viral Injection

All animal experiments were approved by the Experimental Animal Ethics Committee of Nanchang University (protocol code NCU‐LL20180905, approved on September 5, 2018) and conducted in accordance with the Guide for the Care and Use of Laboratory Animals (NIH publication No. 86‐23, revised 1985).

All mice were C57BL/6J background and maintained at 22°C–25°C in 12‐h light–dark cycle. 8‐week‐old ApoE^−/−^ mice were fed with HFD for 8 weeks. Recombinant adenoviruses were administered via tail vein injection every 2 weeks, and miR‐375‐3p antagomir (RiboBio, Guangzhou, China; 50 nmol/20 g body weight) was injected weekly. For tissue collection, all mice were anesthetized with an intraperitoneal injection of pentobarbital sodium (60 mg/kg) and euthanized by cervical disarticulation while the mice were under surgical anesthesia.

### 4.3 RNA Isolation and Quantitative Real‐Time Polymerase Chain Reaction (qRT‐PCR) Analysis

Total RNA was isolated from cells or tissues of mice with BMzol Plus reagent (Bmassay, Beijing, China) according to the manufacturer's instructions. For serum samples of mice, Synthetic *Caenorhabditis elegans* miR‐39 (cel‐miR‐39, RiboBio, Guangzhou, China) at 30 nM was added to each RNA sample for normalization before qRT‐PCR. Reverse transcription was performed with Reverse Transcription reagent (Promega, Madison, WI, USA) according to the manufacturer's instructions. qRT‐ PCR was performed with a Qtower^3^G Real‐Time system (Analytik Jena, Jena, Germany) and 2 × Realtime PCR Super mix (Mei5bio, Beijing, China). The relative expression of mRNA or miRNA was evaluated by the 2^−ΔΔCt^ method and normalized to the expression of GAPDH or U6, respectively. The primers used in this study are listed in Table S3.

### 4.4 MiRNA Mimics/siRNAs Transfection and Adenovirus Infection

For transfection, cells were transfected with miR‐375‐3p mimic, siRNAs, or corresponding negative controls (Scramble) using Lipofectamine 2000 (Thermo Fisher Scientific, Waltham, MA, USA) according to the manufacturer's instructions. The miR‐375‐3p mimic and negative controls were synthesized by GenePharma (Shanghai, China). STX6 and SMAD2 specific siRNAs were obtained from Genebio (Shanghai, China). Sequences are listed in Table S4.

For viral infection assay, cells were infected with pAdeno‐EF1A(S)‐mScarlet‐CMV‐STX6‐3FLAG recombinant adenovirus (OBIO, Shanghai, China) to overexpress STX6 following the manufacturer's guidelines. pAdeno‐EF1A(S)‐mScarlet‐CMV‐3FLAG recombinant adenovirus (OBIO, Shanghai, China) was used as a negative control. The adenovirus was constructed based on the human STX6 gene sequence, which shares high amino acid homology with the murine STX6 protein. Therefore, the same adenoviral vector was used for both in vitro and in vivo experiments.

### 4.5 Western Blot

Total protein was extracted from HUVECs in RIPA buffer (Beyotime, Shanghai, China) containing Protease Inhibitor Cocktail (Roche, Basel, Switzerland) and quantified using a BCA Protein Assay Kit (Beyotime, Shanghai, China). Equal amounts (30 μg) of protein samples were separated by 12% SDS‐PAGE and transferred to polyvinylidene difluoride (PVDF) membrane (Millipore, MA, USA). Membranes were blocked with 5% BSA in TBST and incubated with primary antibodies. After incubation with HRP‐conjugated anti‐rabbit IgG or anti‐mouse IgG secondary antibodies (Proteintech, Wuhan, Hubei, China), membranes were imaged using SuperSignal West Pico PLUS (Thermofisher, Waltham, MA, USA).

The primary antibodies included STX6, GAPDH (Proteintech, Wuhan, Hubei, China), p‐SMAD2, SMAD2 (Cell Signaling Technology, Boston, MA, USA), p15 (Santa Cruz Biotechnology, Dallas, TX, USA). For western blot, all antibodies were used at a dilution of 1: 1000.

### 4.6 Senescence‐Associated β‐Galactosidase Staining

SA‐β‐gal activity was assessed using a Senescence‐Associated β‐Galactosidase Staining Kit (Bmassay, Beijing, China) following the manufacturer's protocol. Briefly, cells were washed twice with PBS, fixed with 4% paraformaldehyde for 5 min, and then incubated with staining solution at 37°C in the dark for 12 h. Positive cells stained blue and were imaged using an inverted microscope (Zeiss Axio, Germany).

### 4.7 Colony Formation Assay

HUVECs were seeded into 6‐well plates at a density of 200 cells per well and cultured for 7 days, with medium replaced every 2 days. Colonies were fixed with 4% paraformaldehyde for 15 min and stained with 0.1% crystal violet for 15 min at room temperature. Colonies containing more than 50 cells were counted. The assay was performed in three independent biological replicates, and the average colony number per well was used for statistical analysis.

### 4.8 Dual Luciferase Reporter Assay

HUVECs were seeded into 24‐well plates (5 × 10^4^ cells per well) and transfected with pMIR‐REPORT luciferase vectors containing the wild‐type or mutant 3′UTR of STX6, along with miR‐375‐3p mimic or scramble oligonucleotides, using Lipofectamine 2000. After 48 h, luciferase activity was measured using the Dual‐Luciferase Reporter Assay System (Promega, Madison, WI, USA).

### 4.9 Immunofluorescence (IF) and Receptor Internalization

Cells or frozen sections (6 μm) were fixed in cold methanol for 10 min and blocked with 1% BSA for 1 h. Samples were incubated overnight at 4°C with primary antibodies, washed three times with PBST, and then incubated with fluorescent secondary antibodies (Invitrogen, Carlsbad, CA, USA) for 1 h at room temperature. Nuclei were counterstained with Hoechst 33342, and images were captured by confocal microscopy.

For TGFBR1 internalization, serum‐starved cells were incubated with TGF‐β1 at 4°C for 30 min to allow ligand–receptor binding, followed by incubation at 37°C for 1 h before fixation. To quantitate TGFBR1 colocalization with EEA1, representative cells were captured by a × 63 lens after IF and the rate of colocalization was analyzed as we previously described (Liu et al. 2013). The primary antibodies used included p‐SMAD2 and EEA1 (Cell Signaling Technology, Boston, MA, USA), TGFBR1 (Santa Cruz Biotechnology, Dallas, TX, USA), MOMA2 (Sigma‐Aldrich, St. Louis, MO, USA), and CD31 (ABCAM, Cambridge, UK). All antibodies were used at a dilution of 1: 100.

### 4.10 Oil Red O Staining

Oil red O staining was used to assess the atherosclerotic lesion in arterial lumina. The whole thoracic‐abdominal aorta or frozen sections (6 μm) of aortic root was fixed with 4% paraformaldehyde, stained with 0.5% oil red O solution for 15 min, and then differentiated in a 60% propylene glycol solution for 5 min. The stained aortas were photographed by digital single lens reflex and sections were imaged by microscope.

For quantitative analysis, the Oil Red O‐stained areas in the aortic root and aortic tree were measured using ImageJ software (National Institutes of Health, USA). In cross‐sectional analysis of the aortic root, the lesion area was manually outlined and expressed as the percentage of Oil Red O‐positive area relative to the total luminal area bounded by the internal elastic lamina (IEL). For en face analysis of the aortic tree, the total lipid‐stained area was measured and expressed as a percentage of the total aortic surface area. All image analyses were performed in a blinded manner by two independent investigators.

### 4.11 Total Cholesterol and High‐Density Lipoprotein Cholesterol Detection

The blood was taken from the eye sockets of mice and left to stand at room temperature for 30 min. The serum was separated by centrifugation at 1500×*g* for 10 min. TC and HDL‐C levels were measured using the cholesterol oxidase–peroxidase method and a homogeneous assay, respectively, on a fully automated biochemical analyzer (Hitachi 7600, Tokyo, Japan) respectively.

### 4.12 Statistical Analysis

All measurement data were expressed as the mean ± SD. Treatment group values were compared with the corresponding control values using GraphPad Prism 8.0 (GraphPad Software). The Shapiro–Wilk test was first applied to assess the normality of the dataset, followed by the Bartlett test to evaluate the homogeneity of variances, assuming the data met the normality assumption. For comparisons between two groups, a two‐tailed Student's *t*‐test was used. For comparisons among multiple groups, either one‐way or two‐way analysis of variance (ANOVA) was performed as appropriate, followed by Tukey's post hoc test. *p* < 0.05 was considered significant for all experiments.

## Author Contributions

**Xiao‐Li Tian**, **Ying Zhu**, and **Zhirui Liu:** conceptualization. **Ying Zhu**, **Zhirui Liu**, **Yiqi Wan**, **Shuangjin Ding**, **Jiankun Liu**, and **Andong Wu:** methodology. **Ximo Dai**, **Jin Zhou**, **Xueer Li**, **Xueting Gong**, and **Man Liu:** investigation. **Ying Zhu**, **Zhirui Liu**, and **Yiqi Wan:** writing original draft. **Xiao‐Li Tian:** supervision and funding acquisition.

## Funding

This work was supported by the Key Program of the National Natural Science Foundation of China, 82330046. National Key Research and Development Program of the Ministry of Science and Technology, China, 2023YFC3603300. Jiangxi Province Key Laboratory of Aging and Disease, 2024SSY07161.

## Disclosure

Permission Statement: All data generated and/or analyzed during this study are permissible.

## Conflicts of Interest

The authors declare no conflicts of interest.

## Supporting information


**Figure S1:** Summary of the miRNA functional screen.

**Figure S2:** Inhibition of miR‐375‐3p attenuates endothelial senescence and atherosclerosis in ApoE^−/−^ mice. (a) Schematic representation of the in vivo experimental design. ApoE^−/−^ mice fed HFD received weekly tail vein injections of miR‐375‐3p antagomir (50 nmol/20 g body weight) or negative control for 6 weeks. (b) Representative Oil Red O staining images and quantification of plaque area in the entire aortic tree. (c) Representative Oil Red O staining images and quantification of lesion size and positive area in the aortic sinus. Scale bar = 200 μm. (d) SA‐β‐gal staining of aortic root sections. Scale bar = 20 μm. Data are presented as mean ± SD; *n* = 3 per group; statistical significance determined by unpaired two‐tailed Student's *t*‐test. **p < 0.01.

**Figure S3:** Screening and validation of STX6 as a miR‐375‐3p target gene. (a) Workflow of 12 candidate target genes selected from 149 predicted targets and RNA‐seq data. (b, c) Luciferase assay of WT (b) and mutant (c) reporters in HEK293A cells transfected with miR‐375‐3p mimic or scramble. (d, e) qRT‐PCR analysis of RIDA (d) and PAFAH1B1 (e) mRNA after siRNA transfection in HUVECs, normalized to GAPDH. (f, g) Representative SA‐β‐gal staining of HUVECs transfected with RIDA (f) or PAFAH1B1 (g) siRNAs. Scale bar = 200 μm. (h) Representative SA‐β‐gal staining of HUVECs infected with Ad‐RIDA and transfected with miR‐375‐3p mimic. Scale bar = 200 μm. Data are presented as mean ± SD; statistical significance determined by unpaired two‐tailed Student's *t*‐test or two‐way ANOVA. *p < 0.05, **p < 0.01, ***p < 0.001.

**Figure S4:** Validation of STX6 overexpression in HUVECs by western blot. Western blot analysis of STX6 protein levels in HUVECs infected with Ad‐STX6 or Ad‐Ctrl, normalized to GAPDH. Data are presented as mean ± SD; statistical significance determined by unpaired two‐tailed Student's *t*‐test. **p < 0.01.

**Figure S5:** Exogenous TGF‐β1 promotes endothelial cell senescence. (a) Representative SA‐β‐gal staining and quantification in HUVECs stimulated with increasing doses of TGF‐β1. Scale bar = 200 μm. (b) Western blot analysis of p15 protein expression in HUVECs stimulated with TGF‐β1. Data are presented as mean ± SD; statistical significance determined by one‐way ANOVA. **p < 0.01, ***p < 0.001.

**Figure S6:** Verification of STX6 overexpression and metabolic changes in ApoE^−/−^ mice. (a) qRT‐PCR analysis of Stx6 expression in aortas of ApoE^−/−^ mice injected with Ad‐Ctrl (*n* = 8) or Ad‐STX6 (*n* = 8), normalized to Gapdh. (b) Immunofluorescence of phosphorylated Stx6 (yellow) and Cd31 (red) in the aortic intima (L) of Ad‐Ctrl (*n* = 6) or Ad‐STX6 (*n* = 6) mice. Nuclei stained with Hoechst (blue). Scale bar = 20 μm. (c–e) Plasma total cholesterol (TC, c) and high‐density lipoprotein cholesterol (HDL, d) levels, and body weight (e), in Ad‐Ctrl (*n* = 8) and Ad‐STX6 (*n* = 8) mice. Data are presented as mean ± SD; statistical significance determined by unpaired two‐tailed Student's *t*‐test. **p < 0.01.

**Data S1:** acel70326‐sup‐0007‐Tables.xlsx.

## Data Availability

All relevant data can be found within the article and its Supporting Information.
