# Supplementary figures and images for: miR‐375‐3p/STX6 Exacerbates Atherosclerosis by Promoting Endothelial Cell Senescence via Activation of TGF‐Beta Signals
Source: Aging Cell. 2025 Dec 10;25(1):e70326. doi: 10.1111/acel.70326 (PMC12741199; doi:10.1111/acel.70326)

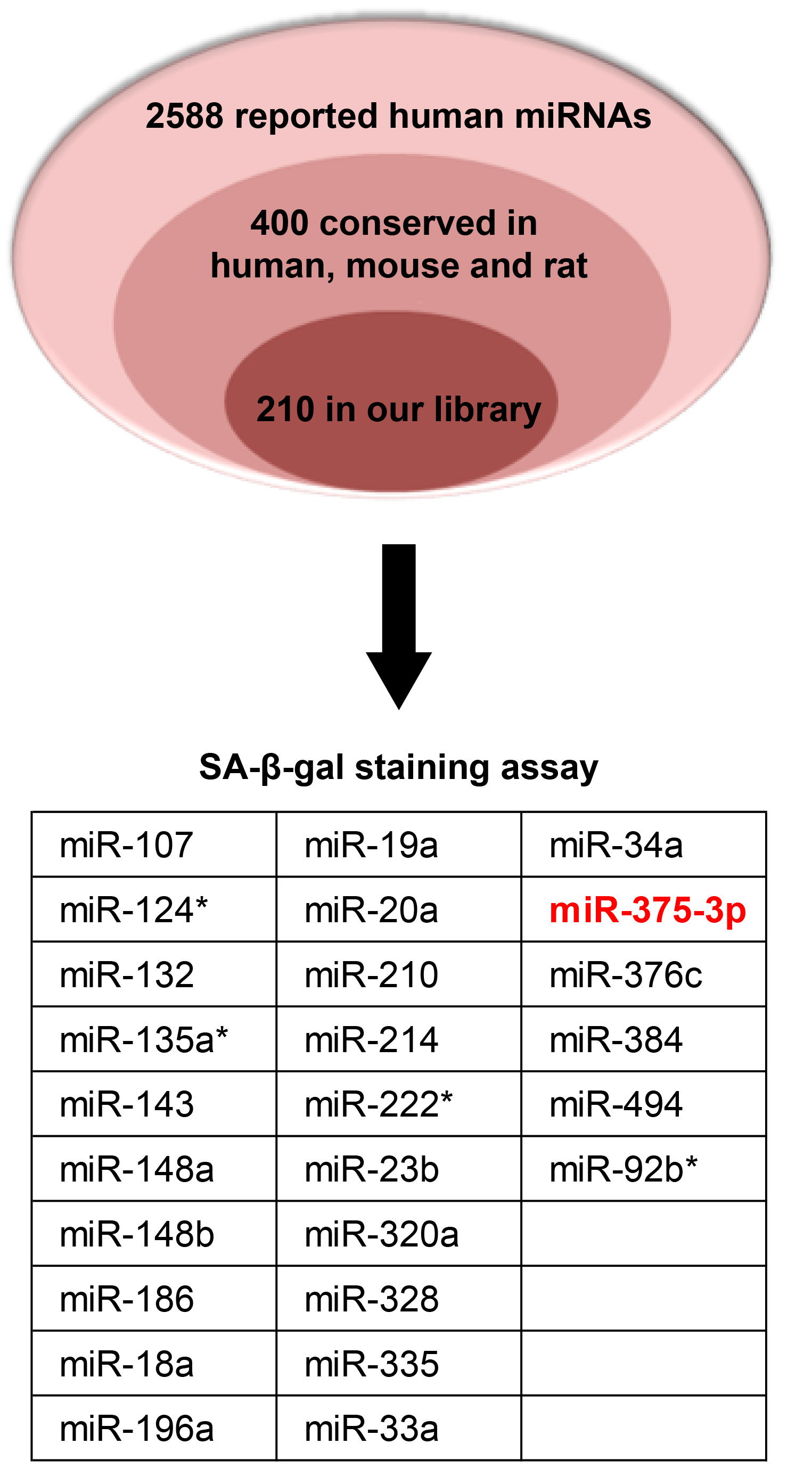

Supplement: Supplementary file 1 — Figure S1: Summary of the miRNA functional screen. [file ACEL-25-e70326-s004.jpg]

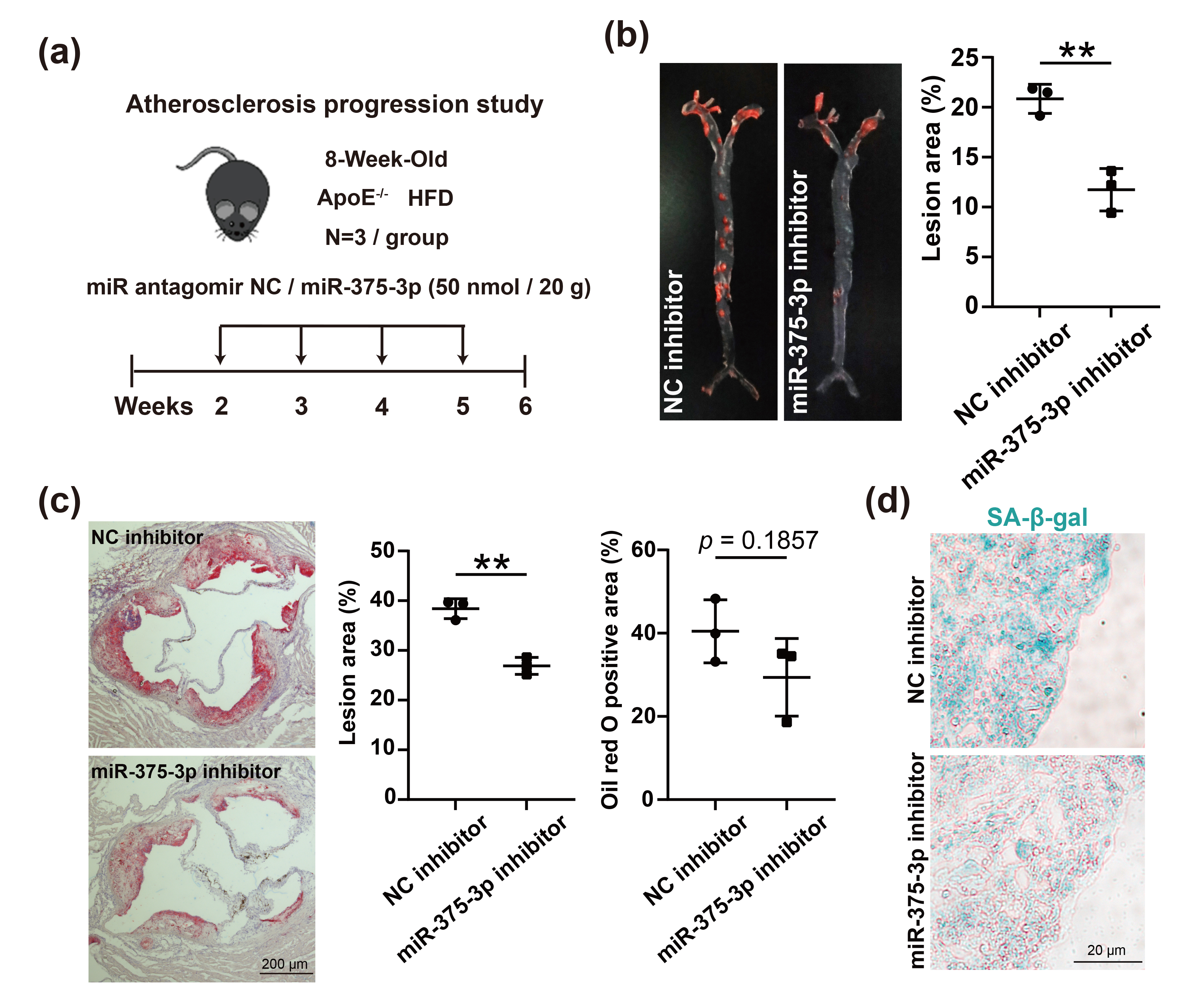

Supplement: Supplementary file 2 — Figure S2: Inhibition of miR‐375‐3p attenuates endothelial senescence and atherosclerosis in ApoE−/− mice. (a) Schematic representation of the in vivo experimental design. ApoE−/− mice fed HFD received weekly tail vein injections of miR‐375‐3p antagomir (50 nmol/20 g body weight) or negative control for 6 weeks. (b) Representative Oil Red O staining images and quantification of plaque area in the entire aortic tree. (c) Representative Oil Red O staining images and quantification of lesion size and positive area in the aortic sinus. Scale bar = 200 μm. (d) SA‐β‐gal staining of aortic root sections. Scale bar = 20 μm. Data are presented as mean ± SD; n = 3 per group; statistical significance determined by unpaired two‐tailed Student's t‐test. **p < 0.01. [file ACEL-25-e70326-s003.jpg]

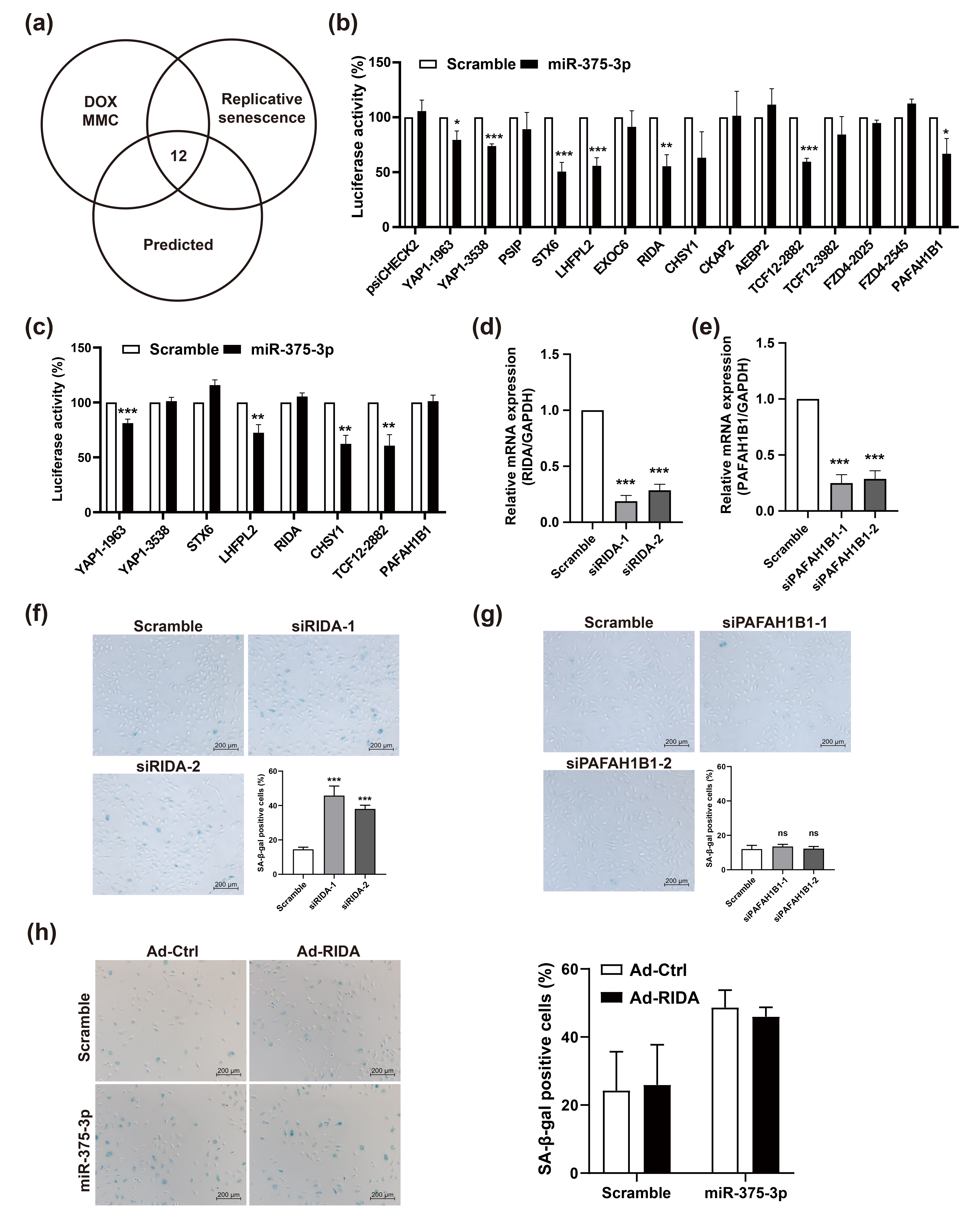

Supplement: Supplementary file 3 — Figure S3: Screening and validation of STX6 as a miR‐375‐3p target gene. (a) Workflow of 12 candidate target genes selected from 149 predicted targets and RNA‐seq data. (b, c) Luciferase assay of WT (b) and mutant (c) reporters in HEK293A cells transfected with miR‐375‐3p mimic or scramble. (d, e) qRT‐PCR analysis of RIDA (d) and PAFAH1B1 (e) mRNA after siRNA transfection in HUVECs, normalized to GAPDH. (f, g) Representative SA‐β‐gal staining of HUVECs transfected with RIDA (f) or PAFAH1B1 (g) siRNAs. Scale bar = 200 μm. (h) Representative SA‐β‐gal staining of HUVECs infected with Ad‐RIDA and transfected with miR‐375‐3p mimic. Scale bar = 200 μm. Data are presented as mean ± SD; statistical significance determined by unpaired two‐tailed Student's t‐test or two‐way ANOVA. *p < 0.05, **p < 0.01, ***p < 0.001. [file ACEL-25-e70326-s006.jpg]

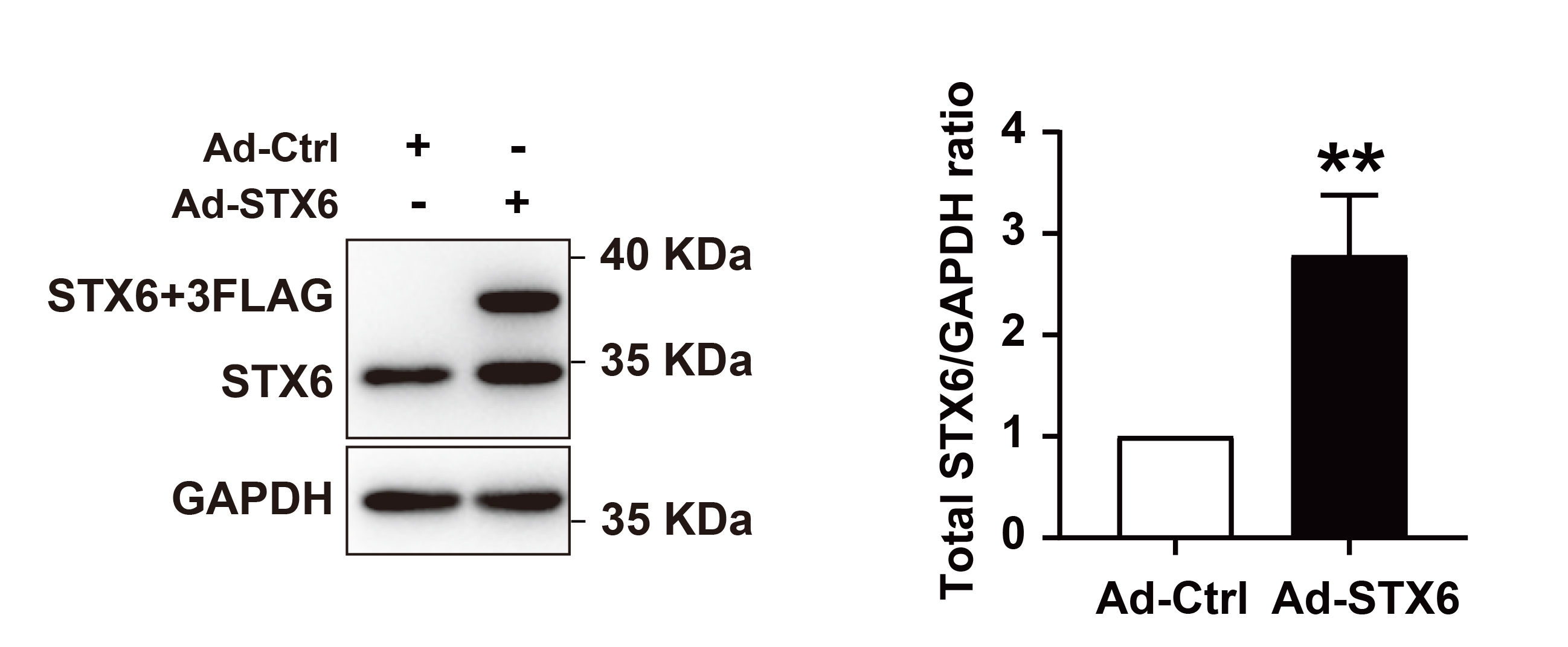

Supplement: Supplementary file 4 — Figure S4: Validation of STX6 overexpression in HUVECs by western blot. Western blot analysis of STX6 protein levels in HUVECs infected with Ad‐STX6 or Ad‐Ctrl, normalized to GAPDH. Data are presented as mean ± SD; statistical significance determined by unpaired two‐tailed Student's t‐test. **p < 0.01. [file ACEL-25-e70326-s005.jpg]

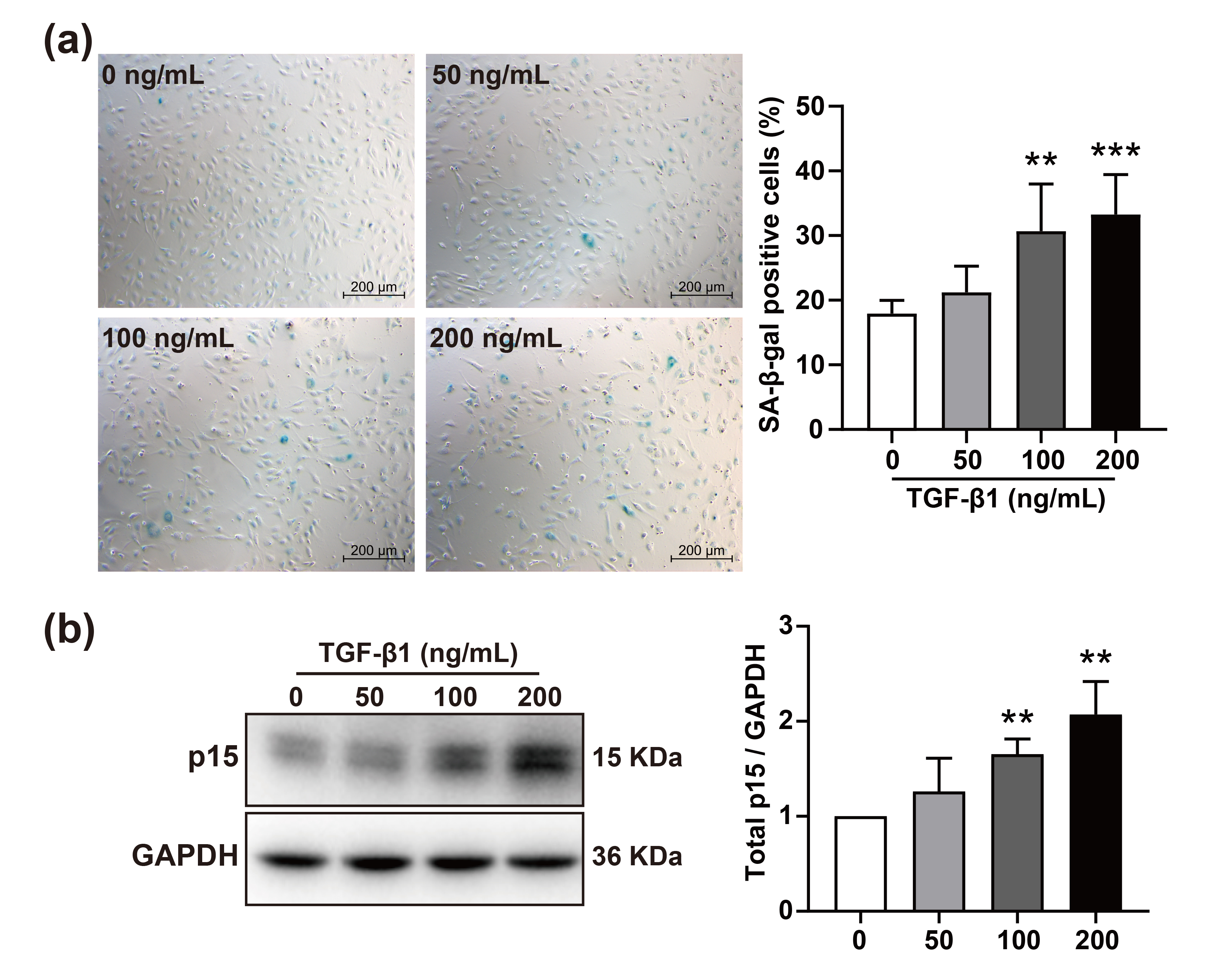

Supplement: Supplementary file 5 — Figure S5: Exogenous TGF‐β1 promotes endothelial cell senescence. (a) Representative SA‐β‐gal staining and quantification in HUVECs stimulated with increasing doses of TGF‐β1. Scale bar = 200 μm. (b) Western blot analysis of p15 protein expression in HUVECs stimulated with TGF‐β1. Data are presented as mean ± SD; statistical significance determined by one‐way ANOVA. **p < 0.01, ***p < 0.001. [file ACEL-25-e70326-s001.jpg]

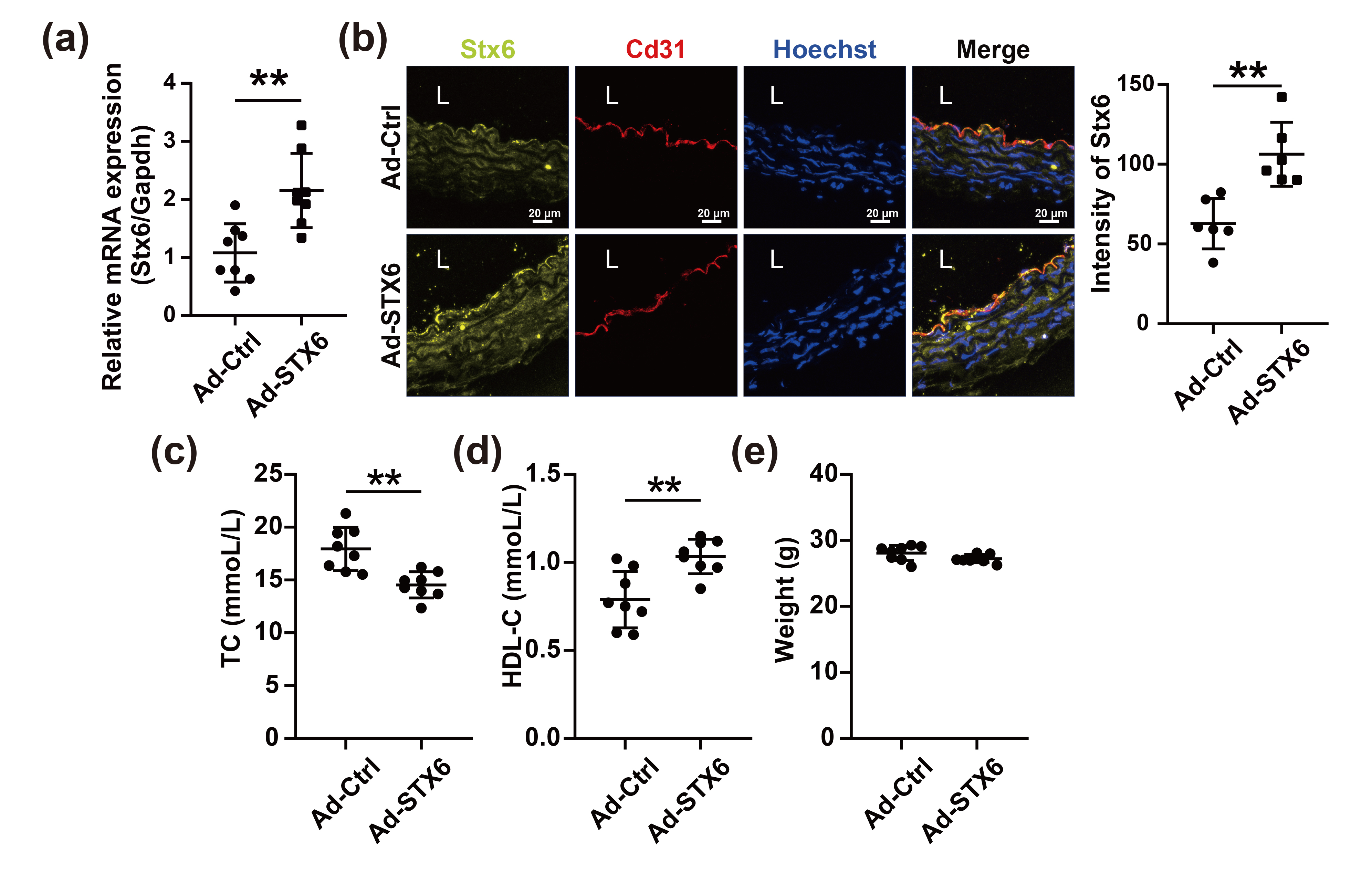

Supplement: Supplementary file 6 — Figure S6: Verification of STX6 overexpression and metabolic changes in ApoE−/− mice. (a) qRT‐PCR analysis of Stx6 expression in aortas of ApoE−/− mice injected with Ad‐Ctrl (n = 8) or Ad‐STX6 (n = 8), normalized to Gapdh. (b) Immunofluorescence of phosphorylated Stx6 (yellow) and Cd31 (red) in the aortic intima (L) of Ad‐Ctrl (n = 6) or Ad‐STX6 (n = 6) mice. Nuclei stained with Hoechst (blue). Scale bar = 20 μm. (c–e) Plasma total cholesterol (TC, c) and high‐density lipoprotein cholesterol (HDL, d) levels, and body weight (e), in Ad‐Ctrl (n = 8) and Ad‐STX6 (n = 8) mice. Data are presented as mean ± SD; statistical significance determined by unpaired two‐tailed Student's t‐test. **p < 0.01. [file ACEL-25-e70326-s002.jpg]
